# Genetic characterization of the HIV-1 reservoir after Vacc-4x and romidepsin therapy in HIV-1-infected individuals

**DOI:** 10.1097/QAD.0000000000001861

**Published:** 2018-05-11

**Authors:** Anni Winckelmann, Vincent Morcilla, Wei Shao, Mariane H. Schleimann, Jesper F. Hojen, Timothy E. Schlub, Paul W. Benton, Lars Østergaard, Ole S. Søgaard, Martin Tolstrup, Sarah Palmer

**Affiliations:** a Centre for Virus Research, The Westmead Institute for Medical Research, The University of Sydney, Sydney, New South Wales, Australia; b The Department of Infectious Diseases, Aarhus University Hospital, Aarhus, Denmark; c Advanced Biomedical Computing Center, Leidos Biomedical Research Inc., Frederick National Laboratory for Cancer Research, Frederick, Maryland, USA; d Institute of Clinical Medicine, Aarhus University, Aarhus, Denmark; e Sydney School of Public Health, Sydney Medical School, The University of Sydney, Sydney, New South Wales, Australia.

**Keywords:** HIV cure, HIV latency, HIV phylogenetics, latency reversing agent, romidepsin, single-genome sequencing, therapeutic HIV vaccine

## Abstract

**Objective:**

Therapeutic HIV-1 immunization followed by latency reversal has been suggested as a strategy to eradicate HIV-1. Here we investigate the phylogenetic composition of the HIV-1 regions targeted by the therapeutic HIV-1 peptide vaccine Vacc-4x in participants in a clinical trial.

**Design:**

Seventeen participants on suppressive antiretroviral therapy were vaccinated with six doses of Vacc-4x followed by three doses of romidepsin. Seven study participants were selected for sequencing analysis. All participants underwent an analytical treatment interruption.

**Methods:**

Single-genome/proviral sequencing of the p24-RT region was performed to genetically characterize proviral DNA, cell-associated RNA and outgrowth viruses during therapy as well as plasma HIV-1 RNA during an analytical treatment interruption.

**Results:**

There were no changes in cell-associated HIV-1 RNA (*P* = 0.83) and DNA (*P* = 0.09) diversity over the course of the study and no difference between cell-associated HIV-1 RNA and DNA diversity (*P* = 0.32). Only one participant showed signs of potential vaccine-related selection in the rebounding plasma virus. In five of seven participants, we identified human leukocyte antigen-specific cytotoxic T lymphocytes (CTL) epitopes containing nonsilent mutations in 100% of the sequences.

**Conclusion:**

We detected no evidence of selective immune pressure reflected in proviral diversity or by occurrence of specific mutation in the vaccine-targeted epitopes. Preexisting CTL epitope mutations may affect the potency of this therapeutic vaccine. This highlights the challenges of developing effective HIV-1 therapeutic vaccines.

## Introduction

Integrated proviruses are a barrier to cure HIV-1 and can give rise to viral rebound when antiretroviral therapy (ART) is interrupted. One strategy to eradicate HIV-1 aims to reactivate viral transcription in latently infected cells with latency reversing agents (LRAs). Several trials with histone deacetylase inhibitors have provided proof of concept that viral reactivation is achievable [1–6]. This eradication strategy heavily relies on subsequent elimination of the reactivated cells by immune-mediated killing by cytotoxic T lymphocytes (CTL). Boosting the CTL response prior to latency reversal has been suggested as a way to overcome the waning T-cell mediated immunity observed in many long-term ART-treated individuals. However, the CTL response may be compromised by the presence of CTL epitope-mutated viruses in individuals treated during chronic HIV-1 infection [7].

A recent clinical trial, REDUC part B, assessed the combination of a peptide-based therapeutic HIV-1 vaccine (Vacc-4x) and the LRA romidepsin. This combinatorial approach resulted in reductions in the latent HIV-1 reservoir [8] which were associated with general positive CD8^+^ T-cell proliferation *ex vivo*[9]. Vacc-4x is comprised of four modified peptides corresponding to conserved regions within HIV-1 p24 and is delivered intradermally with recombinant human granulocyte macrophage colony-stimulating factor (rhuGM-CSF) as local adjuvant. However, any qualitative antigen-specific vaccine-induced effects remain elusive.

In this study, we performed single-genome/proviral sequencing on circulating proviruses, stimulated outgrowth viruses and rebound viruses to investigate the genetic composition of the infecting virus with respect to the four gag domains targeted by the therapeutic vaccine Vacc-4x in participants enrolled in the clinical vaccine trial REDUC part B. We hypothesized that any CTL immune-mediated effects would result in a selection of mutations in the Vacc-4x-targeted domains in the rebounding virus during an analytical treatment interruption (ATI) and a decrease in genetic diversity of cell-associated HIV-1 RNA and DNA following the intervention.

## Methods

### Study design

The current study is a follow-on study to the REDUC part B clinical trial. In this trial, 17 HIV-1-infected individuals on long-term suppressive ART received a series of six intradermal immunizations over 12 weeks (at 0, 1, 2, 3, 11 and 12 weeks) with Vacc-4x (Bionor Pharma, Oslo, Norway) and rhuGM-CSF (Genzyme, Cambridge, Massachusetts, USA) as adjuvant. The immunization phase was followed by an activation phase with three intravenous infusions of romidepsin (Celgene, Summit, New Jersey, USA) once weekly for 3 weeks while maintaining ART. Subsequently 16 participants underwent an ATI. Detailed clinical trial design, patient characteristics and total HIV-1 DNA quantitation (listed in Table 1) have been published previously [8]. Vacc-4x is investigational and romidepsin is not labeled for use in HIV infection.

### Ethics statement

The study was approved by the Danish Health and Medical Authorities as well as the Danish Data Protection Agency. The study was approved by the National Committee on Health Research Ethics (#M-2013-364-13) in accordance with the principles of the Helsinki Declaration. Each participant provided written informed consent prior to any study procedures. The trial is registered at http://clinicaltrials.gov (NTC02092116).

### Participants and samples

A subset of seven study participants were selected for single genome/proviral sequencing analysis, all participated in the ATI. The participants were selected to cover the span of changes in proviral DNA. Three participants with a substantial drop in total HIV-1 DNA (participant identification (ID)23, 36 and 43 with a decrease of −44, −72 and −86%, respectively) and four with limited change in total HIV-1 DNA (ID24, 25, 27 and 29 with changes in proviral DNA content of +55, −8, −19 and −18%, respectively). Second, patients with viral outgrowth assay (VOA) culture material were prioritized. The participant characteristics are compiled in Table 1. We analyzed cell-associated HIV-1 RNA and DNA from CD4^+^ T cells at baseline, two time points during the immunization phase, two time points during romidepsin therapy and at a follow-up visit (refer to Figure of study design, Supplemental Digital Content 1). Baseline samples were obtained 3 weeks prior to the first Vacc-4x immunization. The samples from the immunization phase were obtained immediately before the fourth immunization and 3 weeks after the sixth immunization and were designated immunization time points 1 and 2. The samples from the activation phase were obtained 4 h after the second and third romidepsin infusions and were designated romidepsin time points 1 and 2. Follow-up samples were obtained 6 weeks after the third romidepsin infusion. Viremic plasma samples were obtained during the ATI just prior to reinitiation of ART, viral loads ranging between 300 and 5000 copies/ml. From participant 24, two ATI time points were analyzed. The VOA was performed on resting CD4^+^ T cells from baseline, 2 weeks after the sixth immunization and follow-up [8]. We obtained VOA supernatants from wells that were tested HIV-1 positive using the TZM-bl luciferase reporter assay.

### Nucleic acid extraction and complementary DNA synthesis

Plasma was obtained following centrifugation of blood ethylenediaminetetraacetic acid-collection tubes. Peripheral blood mononuclear cells (PBMCs) were separated by gradient centrifugation in cell preparation tubes. CD4^+^ T cells were isolated from PBMCs using a CD4^+^ T-cell negative isolation kit and magnetic-activated cell sorting columns (Miltenyi Biotec, Teterow, Germany; purity >95%). One million CD4^+^ T cells were lysed in QIAgen lysis (RLT) buffer, and the lysates were stored at −80 °C until the RNA and DNA were extracted using the QIAgen AllPrep DNA/RNA Mini Kit (Cat.no. 80204, QIAgen, Melbourne, Australia). During RNA purification, RNA columns were treated twice with QIAgen RNase-Free DNase. Plasma HIV-1 RNA for sequencing was extracted from 2 to 3 ml of plasma by ultracentrifugation and a guanidinium-based method, including a prespin to sediment potential cell remnants [10]. HIV-1 RNA was extracted from 10 to 100 μl VOA supernatant using the same method as the plasma samples, but without the prespin. From cell-associated HIV-1 RNA, plasma HIV-1 RNA and VOA HIV-1 RNA, we generated complementary DNA using the Superscript III (Invitrogen by Thermo Fisher Scientific) cDNA synthesis kit and a gene-specific primer for p24-RT according to the manufacturer's instructions. See Supplemental Digital Content 2 for primers and PCR conditions.

### Single-genome/proviral sequencing

We performed single-genome/proviral sequencing of a 2.1-kb region spanning HIV-1 p24 to RT [11–14]. Single HIV-1 molecules were sequenced using Sanger sequencing *(Australian Genome Research Facility, Sydney Australia)*. See Supplemental Digital Content 2 for PCR and sequencing primers and PCR conditions.

### Phylogenetic analysis

Contigs were generated from the raw sequencing data using an in-house computer program written in Perl scripting language (available upon request). Vigorous automated and manual quality-control parameters were used to eliminate low-quality sequences prior to and following the generation of the contigs. From each positive VOA well, we obtained five to nine single genomes. The median diversity of the genomes obtained from one well was 0.067% average pairwise distance (range 0.011–0.669%). One consensus sequence was generated for each positive VOA well for further phylogenetic analysis. Multiple alignment files were created for each participant using MUSCLE [15]. Phylogenetic analysis of the sequences from all seven participants formed individual clades with no interparticipant mixing, except for ID24 and 25, who are a known transmission pair (refer to Figure, Supplemental Digital Content 3). Defective viruses were characterized using the Los Alamos HIV-1 Database Hypermut tool (www.hiv.lanl.gov) to screen for sequences containing G-A hypermutations and by screening the amino-acid sequences for premature stop codons. Sequences assigned with a *P* value less than 0.05 by the Hypermut tool were defined as hypermutated. Defective viruses were excluded, and the remaining sequences were used to construct maximum likelihood phylogenetic trees using Molecular Evolutionary Genetics Analysis version 6.0 (MEGA6; https://www.megasoftware.net/webhelp/contexthelp_hc/hc_citing_mega_in_publications.htm) [16]. An appropriate model for nucleotide substitution was determined for each phylogenetic tree using MEGA model finder. The models used to generate the phylogenetic trees in this study included the following: the general-time reversible model and the Hasegawa–Kishino–Yano model incorporating gamma distributed (gamma category 4) and/or invariant sites where appropriate. Our heuristic tree search strategy used the nearest neighbor interchanges branch swapping algorithm. Branch support was inferred using 1000 bootstrap replicates. The phylogenetic trees were visualized and annotated using the ggtree package version 1.8.1 in R programming language [17]. Measurements of genetic HIV-1 diversity (average pairwise distance, APD) of HIV-1 DNA or RNA sequences were calculated after excluding defective sequences in MEGA 6.0. Samples with fewer than five sequences were excluded from analyses to reduce bias, based on a simulation of sampling as reported previously [11]. Identical and similar sequences were identified using the number of differences model in MEGA 6.0. All intact sequences from the study are available in GenBank (MG770940-MG772098).

### Epitope mutation analysis

To address the presence of possible escape mutations in the four domains targeted by Vacc-4x, we identified CTL epitopes within the target regions of the four vaccine-peptides; Vacc-10 (HXB2 p24 site 35–57: PEVIPMFSALSEGATPQDLN), Vacc-11 (HXB2 p24 site 120–137: NNPPIPVGEIYKRWIILG), Vacc-12 (HXB2 p24 site 132–152: RWIILGLNKIVRMYSPTSILD) and Vacc-13 (HXB2 p24 site 203–222: KALGPAATLEEMMTACQGVG) based on human leukocyte antigen (HLA) type using the Los Alamos database tool Epitope Location Finder (www.hiv.lanl.gov). HLA typing was performed on PBMCs at the Department of Clinical Immunology, Aarhus University Hospital (refer to Table, Supplemental Digital Content 4). We searched for identifiable variants in HLA-matched CTL epitopes using the Los Alamos Epitope Variant and Escape Mutation Database (www.hiv.lanl.gov).

### Intracellular cytokine staining and viral inhibition assay

To assess HIV-specific immune responses, we performed intracellular cytokine staining for IFN-γ, TNF-α and IL-2 on PBMCs after stimulation with an HIV-1 Gag peptide pool. An autologous viral inhibition assay was performed by infection of target CD4^+^ T cells with the laboratory adapted HIV-1 HXB2 strain followed by addition of CD8^+^ T cells. Both methods have been described in detail previously [9].

### Statistics

Sequences from both time points during the immunization phase were pooled for analysis. Both time points during the romidepsin treatment were also pooled for analysis. A difference in APD across time points was tested using a linear mixed effects model with fixed effect for time point, and a random effect for the intercept across participants. To test for a difference between APD in DNA and RNA, a linear mixed effect model with fixed effects for time points and DNA/RNA and a random effect for intercept across participants was carried out. Linear mixed models were fitted with function *lme* from the *nlme* package [18] and *P* values were calculated with a chi-squared test on an analysis of deviance (function *Anova* in package *car*) [19].

Regular multiple linear regressions with fixed effects for participant were also carried out and drew similar conclusions. There was no evidence for interaction between any of the predictor variables. Statistics were carried out in *R* version 3.4.2 (https://stat.ethz.ch/pipermail/r-help/2008-May/161481.html) [20].

## Results

### Vacc-4x and romidepsin does not impact proviral diversity

**Fig. 1:**
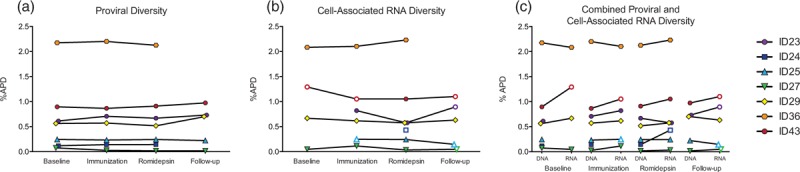
Vacc-4x and romidepsin does not impact proviral diversity.

**Fig. 2:**
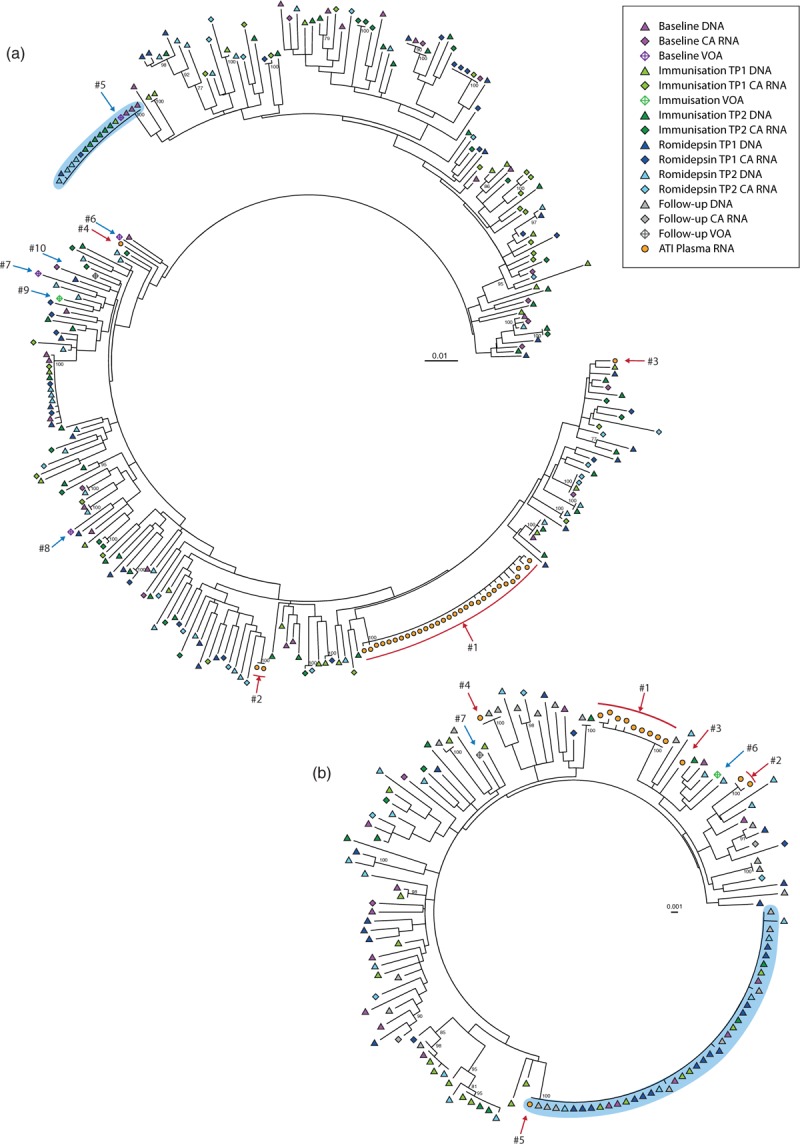
HIV-1 viral outgrowth during antiretroviral therapy and rebound viremia originate from genetically different proviruses.

Enhanced CTL-killing of virus-producing cells by therapeutic vaccination would restrict the genetic diversity of cell-associated HIV-1 RNA and DNA postvaccination. In contrast, if therapeutic vaccination does not enhance CTL-killing of virus-producing cells activated by romidepsin, the genetic diversity of cell-associated HIV-1 RNA and DNA will be similar post romidepsin therapy. To genetically characterize intracellular HIV-1 RNA and DNA post Vacc-4x and romidepsin therapy, we sequenced cell-associated HIV-1 RNA and DNA from peripheral CD4^+^ T cells immediately prior to the first immunization, twice during the immunization phase, twice during romidepsin administration, and once following romidepsin administration. A total of 1160 p24-RT sequences were analyzed (refer to Table, Supplemental Digital Content 5). Using average pairwise distance, we calculated the genetic diversity for all HIV-1 DNA sequences from each participant at each time point and observed no changes in DNA genetic diversity during the intervention (*P* = 0.09, Fig. 1a). The genetic diversity of cell-associated HIV-1 RNA also remained constant throughout the treatment protocol (*P* = 0.83, Fig. 1b). In fact, the average change in HIV-1 genetic diversity for each participant across all time points (within DNA or RNA) was at most 0.244%, with the mean APDs ranging from 0.03 to 2.2% across all patients (RNA and DNA). A comparison of the genetic diversity of cell-associated HIV-1 RNA and DNA sequences revealed no significant difference in average pairwise distance within each participant between the cell-associated HIV-1 RNA and DNA sequences collected at baseline, during the immunization, during romidepsin administration, or at follow-up (*P* = 0.32) (Fig. 1c). In addition, phylogenetic analysis revealed that cell-associated HIV-1 RNA sequences were distributed throughout the phylogenetic tree and intermingled with their corresponding HIV-1 DNA sequences (Fig. 2 and Supplemental Digital Content 6–10).

Average pairwise distance of intact p24-RT sequences from (a and c) proviral HIV-1 DNA and (b and c) cell-associated HIV-1 RNA at study baseline, during the immunization phase, during romidepsin administration and at follow-up. The open symbols indicate that the average pairwise distance is based on less than five sequences and these time points are not included in the statistical analysis. There was no evidence for a change in average pairwise distance across collection times for DNA (*P* = 0.09) or RNA (*P* = 0.83). There was no evidence for a difference in average pairwise distance between DNA and RNA (*P* = 0.32). %APD, percent average pairwise distance.

**Fig. 3:**
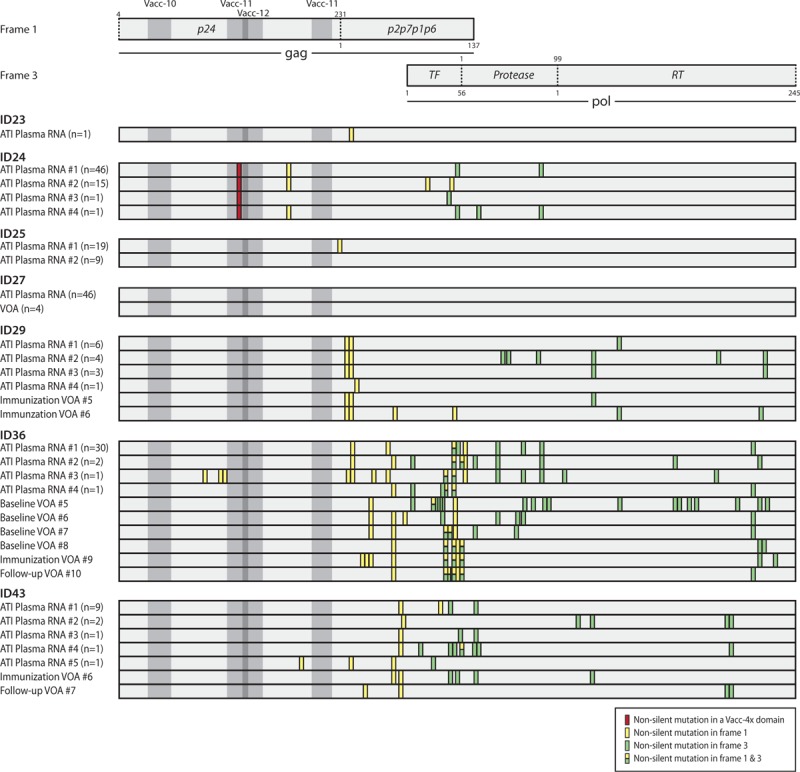
Mutations primarily occurred outside the Vacc-4x-targeted domains.

Maximum likelihood phylogenetic trees of p24-RT sequences for (a) participant 36 and (b) participant 43. Expansions of proviral sequences, which are identical to a viral outgrowth assay or an analytical treatment interruption sequence are highlighted in blue. Red arrows indicate analytical treatment interruption plasma RNA sequences, blue arrows indicate viral outgrowth assay sequences. The numbering of viral outgrowth assay and analytical treatment interruption sequences refer to the numbering in Fig. 3. ATI, analytical treatment interruption; CA, cell-associated; TP, time point; VOA, viral outgrowth assay.

### Nonsilent mutations were located outside the Vacc-4x domains

We hypothesized that any CTL immune-mediated effects would result in a selection of mutations in the Vacc-4x-targeted domains in the rebounding virus. Phylogenetic analysis revealed that in most instances, the replication-competent viruses from the VOA and ATI differed from the proviral pool (Fig. 2 and Supplemental Digital Content 6–10). To investigate whether these differences were due to selection of escape mutations in the Vacc-4x domains, we mapped all nonsilent mutations in VOA and ATI plasma RNA consensus sequences and compared this to the majority sequence of the proviral compartment (Fig. 3). We only identified one instance of a Vacc-4x domain-related nonsilent mutation. From participant 24 in the Vacc-12 domain, the 43 proviral sequences analyzed contained 128E and 128D at a frequency of 88 and 12%, respectively (of note 128D was detected both at baseline and during romidepsin therapy), whereas 100% of the 63 ATI plasma RNA sequences were 128D. Overall, the Vacc-4x domains were conserved compared with the other parts of the region sequenced, as evident in Fig. 3.

Sequence map of nonsilent mutations in the analytical treatment interruption and viral outgrowth assay sequences. A site was mapped when an amino acid in the analytical treatment interruption/viral outgrowth assay consensus sequence was different to the majority of the corresponding proviral pool for each participant. Sites are colored according to their position. Gray boxes indicate the location of the Vacc-4x-targeted domains. The numbering of sequences refers to the numbering of viral outgrowth assay/analytical treatment interruption sequences in the corresponding phylogenetic trees (Fig. 2 and Supplemental Digital Content 6–10). *N* indicates the number of analytical treatment interruption sequences, that each consensus sequence was based on. ATI, analytical treatment interruption; RT, reverse transcriptase; TF, transframe peptide; VOA, viral outgrowth assay.

### Vaccine domains contain preexisting mutations

**Fig. 4:**
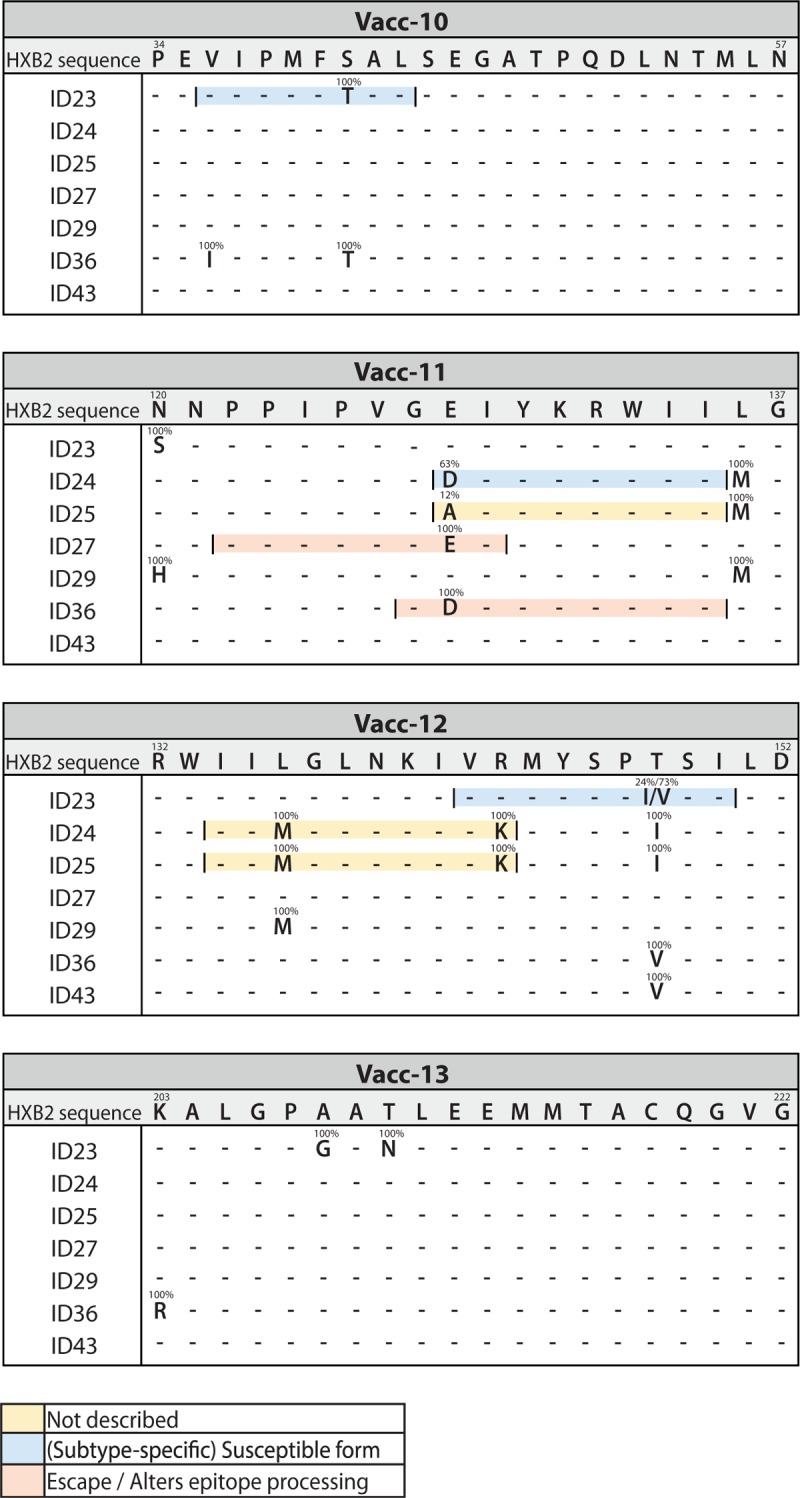
Vacc-4x domains contained preexisting mutations.

To characterize the regions targeted by the vaccine, we identified CTL epitopes within the four Vacc-4x domains based on each participant's HLA type (Fig. 4). Identified variants were classified as the following: an observed variant; a (subtype specific) susceptible form; alters epitope processing or documented escape according to the Los Alamos Epitope Variant and Escape Mutation Database or not described if the variant was not present in the database. Five out of the seven participants had mutations in a HLA-matched CTL epitope that were present in 100% of the sequences obtained, including participant 27, who was treated during acute infection and had a low viral diversity of 0.1% APD. Two participants (ID29 and 43) did not have preexisting mutations in any of the four Vacc-4x domains. Notably, the majority of the preexisting mutations were in Vacc-11 and Vacc-12, and we identified no preexisting or evolving mutations in Vacc-13 in any patient.

For each participant, the Vacc-4x domains are shown. (A) ‘–’ indicates agreement with the HXB2 sequence. Any differences from the HXB2 amino acid sequence are indicated by a letter of the amino acid present. The percentages above the amino acids indicate the number of all obtained sequences from each participant which have the indicated amino acid at this site. Mutated cytotoxic T lymphocytes epitopes based on each individual's HLA type are highlighted and colored based on the type of variant. For ID27 in vacc-11, the highlighted epitope differed from HXB2, and the 128E was defined as mutated. HLA, human leukocyte antigen; ID, participant identification.

### HIV-1 viral outgrowth during antiretroviral therapy and rebound viremia originate from genetically different proviruses

To investigate the origins of replication-competent viruses, we compared stimulated outgrowth viruses to rebounding plasma viruses and the proviral reservoir. VOA was performed during suppressive ART at baseline, during the immunization phase and at follow-up. We obtained sequences from HIV-1 positive wells from four participants. One participant (ID27) had a very low viral diversity of 0.1% and was excluded from this analysis (Figure, Supplemental Digital Content 9). In the remaining three participants (ID29, 36 and 43), we obtained VOA sequences from 10 independent cultures (refer to Table, Supplemental Digital Content 5). In participant 36, we identified a viral outgrowth sequence from baseline that was identical to an expansion of 14 identical proviruses sampled from four time points as well as one cell-associated RNA sequence sampled during romidepsin therapy (Cluster #5, Fig. 2a). In participant 43, we identified an ATI plasma HIV-1 RNA sequence that was identical to an expansion of 36 identical proviruses sampled from all six time points (Cluster #5, Fig. 2b). Notably, this expansion did not match any HIV-1 cell-associated RNA sequences. Overall, there were no instances of genetic similarities between the stimulated outgrowth viruses and rebound viremia.

### No association between HIV-specific immune responses, cytotoxic T lymphocyte epitopes and reservoir changes

To supplement the viral sequence analysis, we have listed both IFN-γ production in CD8^+^ effector memory cells in response to HIV-1 gag overlapping peptide pool as well as functional ex-vivo measurements of CD8^+^ T-cell viral inhibitory capacity (Table 1). There were no significant changes in these seven participants (as reported previously when including all trial participants) [9]. We did not observe any associations between changes in total HIV-1 DNA, the magnitude or changes of the HIV-specific immune responses, and the presence of mutations in CTL epitopes.

## Discussion

Here we sequenced proviruses and rebounding viruses during and after a novel combination treatment using a HIV-1 therapeutic vaccine and an LRA *in vivo*. We report that there were no changes in cell-associated HIV-1 RNA and DNA diversity over the course of the study. Only one participant showed signs of potential vaccine-related selection in the rebounding plasma virus. Furthermore, in five of seven participants, we identified preexisting mutations in HLA-specific CTL epitopes. Collectively, this study did not find evidence for a vaccine-induced immune selective pressure on the rebounding viruses in the ATI following the intervention.

We previously reported a significant decrease in total HIV-1 DNA over the course of the REDUC part B clinical trial. For the analysis reported here, seven individuals were included some of which had proviral DNA reductions of 86 and 78% (ID43 and 36, respectively). However, when studying the proviral DNA diversity, we observed no change in any participant. Furthermore, we did not find a decrease in diversity of the transcribed RNA during romidepsin reactivation either. Similar to previous observations, we found that intracellular HIV-1 RNA and DNA sequences intermingled within the phylogenetic tree indicating a broad activation of proviral expression, which also indicates Vacc-4x did not reduce the level of transcriptionally active and/or virus-producing cells [11,14]. Taken together, these observations indicate that the elimination of HIV-infected cells that did occur was not specifically targeting a subset of immune-susceptible proviruses or transcriptionally active and virus-producing cells. However, it is possible that a selective reduction of the dominant viral variants would not impact the overall genetic diversity of the sequences that were analyzed.

Deng *et al.*[7] previously reported that the majority of proviruses from HIV-infected individuals treated during chronic infection contain CTL escape mutations in immunodominant epitopes. We observed mutations in HLA-matched CTL epitopes in the four vaccine-targeted regions prior to immunization in the majority of participants (five out of seven). Consistent with the concepts proposed by Deng *et al.*, this may pose a major barrier to generating vaccine-induced CTL-responses with a significant impact on the majority of the viral quasispecies. Furthermore, the data presented here specifically questions the relevance of CTL-targeting by Vacc-4x vaccination to regions supposedly conserved. The two participants (ID29 and 43) with no preexisting mutations in the Vacc-4x-targeted region did not develop genetic changes in vaccine-targeted regions of the virus suggestive of immune selective pressure and did not appear to have any benefit on time to rebound during the ATI.

The absence of amino acid mutations different from the majority sequence in the proviral reservoir within the Vacc-4x-targeted domains in the treatment interruption sequences indicates that any immune-pressure was targeted toward other regions of the genome. The only case where the rebound virus contained nonsilent amino acid mutations, that were different from the proviral majority variant, was observed in ID24 in the Vacc-11 region. However, the variant dominating the rebounding viruses (Vacc-11 128D) was detected at 12% frequency in the proviral pool; thus, the occurrence of this rebound virus could have occurred by chance – and not a result of immune-mediated selection pressure. Of note, this participant did not experience a drop in total HIV-1 DNA and had no qualitative increase in the measures of HIV-specific immunity, but interestingly, the virus had the longest doubling time indicative of slower growth kinetics.

We identified expansions of genetically identical proviruses and transcribed RNA that were identical to an ATI or a VOA sequence. This emphasizes that genetically identical proviruses arising from the clonal proliferation of infected cells can be transcriptionally activated by romidepsin and have the capacity to produce virions [21]. However, we did not find overlap between the stimulated outgrowth and treatment interruption sequences which both represent replication-competent viruses. The absence of overlap suggests that either activation of a replication-competent provirus is a unique, random event originating from a very large total pool of potential replication-competent virus or that the rebound virus originates from a pool not sampled by purifying resting CD4^+^ T cells from blood. In this trial, VOA was performed on resting CD4^+^ T cells, and studies have suggested an enrichment of the HIV-1 reservoir in the HLA-DR+ subset of CD4^+^ T cells [22]. The use of resting memory T cells for VOA may limit the capacity to identify relevant replication-competent viruses capable of causing rebound viremia.

There are some limitations to our study. First, we only analyzed a subset of the participants enrolled in the clinical trial. However, those seven selected participants allowed us to compare the genetic composition of HIV-1 DNA and RNA between those participants whose HIV-1 DNA levels dropped during the clinical trial versus those whose did not. Second, for some time points, we generated a small number of HIV-1 RNA sequences. As a result, we limited our genetic diversity calculations to include samples from which we obtained five sequences or more to reduce bias. Finally, single-genome sequencing has some limitations. We were able to identify defective env sequences, but those genomes considered genetically intact in the env region may contain defects in other genomic regions we did not sequence.

In summary, this study shows that a combination treatment of the therapeutic vaccine Vacc-4x and the LRA romidepsin did not result in a selective immune pressure reflected either in the proviral diversity or by occurrence of specific mutations in the vaccine-targeted epitopes. The overall preexisting mutations in CTL epitopes in a very conserved region of gag, even in an HIV-infected individual treated during acute infection, emphasizes the challenges of developing an effective therapeutic T-cell vaccine based on conserved epitopes.

## Acknowledgements

We acknowledge the participation and commitment of the study participants, which made the study possible. A.W. designed and performed experiments, analyzed data and wrote the article. V.M., J.F.H. M.H.S., P.W.D designed and performed experiments and analyzed data. W.S. prepared sequences for analysis. T.E.S. performed the mixed-effects model statistical analyses and contributed to article editing. L.Ø. and O.S.S. designed the study, provided samples and assisted with article preparation. M.T. designed the study, provided samples, supervised the analyses and edited the article. S.P. designed the study, supervised the work performed and edited the article. All authors have read and accepted the final article.

The current work was supported by the Delaney AIDS Research Enterprise (DARE) to Find a Cure (grant numbers 1U19AI096109, 1UM1AI126611-01), the NIH: National Institutes of Allergy and Infectious Diseases grant 1 R21 AI 134204-01 A1, the Australian National Health and Medical Research Council (NHMRC) (grant number APP1061681) and the Australian Centre for HIV and Hepatitis Virology Research (ACH2). L.Ø., M.T. and O.S.S. were supported by a grant from the Danish Council for Strategic Research (grant number 0603-00521B).

### Conflicts of interest

Bionor Pharma sponsored the clinical study from which these samples were derived and contributed to the design of the clinical trial. The funders had no role in data collection and analysis, decision to publish or preparation of the article. L.Ø. is a member of Bionor Pharma's clinical advisory board. The remaining authors declare no competing financial interests.

## Supplementary Material

Supplemental Digital Content

## Figures and Tables

**Table 1:** Study participant characteristics.

						Total HIV-1 DNA			Viral inibition			Intracellular cytokine staining			Analytical treatment interruption	
ID	Sex	Age	Time from HIV-1 diagnosis to ART initiation	Time with HIV-1 RNA <50 copies/ml	Baseline CD4^+^ cell count	Base-line	IMM TP2	Fold diffe-rence	Base-line	IMM TP2	Follow-up	Base-line	IMM TP2	Follow-up	Time to rebound	Virus doubling time
		Years	Months	Months	Cells/μl	Copies/10^6^ CD4^+^ T cells			Log p24 decrease			%IFN-γ+ in CD8^+^ EM			Days	
23	Male	47	16	98	890	3360	2211	0.66	0.62	0.50	0.79	0.006	0.011	0.003	7	0.97
24	Male	52	21	30	860	455	704	1.55	1.51	1.42	nd	0.203	0.121	0.137	21	2.59
25	Female	55	22	31	940	628	579	0.92	1.63	1.60	2.09	3.01	2.50	5.13	42	1.98
27	Male	32	1	24	780	1077	871	0.81	0.86	0.56	0.68	1.42	1.0	1.22	14	1.28
29	Male	39	10	51	610	2839	2342	0.82	nd	nd	nd	0.174	nd	0.146	11	1.17
36	Male	56	116	144	570	5737	1587	0.28	0.33	0.31	0.57	0.082	0.100	0.093	14	0.94
43	Male	57	44	70	730	71	10	0.14	0.48	0.25	0.51	0.078	0.057	0.061	6	0.79
Median		52	21	51	780	1077	871	0.81	0.74	0.53	0.68	0.174	0.111	0.137	14	1.17

ART, antiretroviral therapy; EM, effector memory; ID, IFN, interferon; IMM TP2, immunization time point 2; nd, not determined.
