# Supplementary material for: Genetic characterization of the HIV-1 reservoir after Vacc-4x and romidepsin therapy in HIV-1-infected individuals
Source: AIDS. 2018 May 11;32(13):1793–802. doi: 10.1097/QAD.0000000000001861 (PMC6072375; doi:10.1097/QAD.0000000000001861)
Supplement: Supplemental Digital Content [file aids-32-1793-s001.pdf]

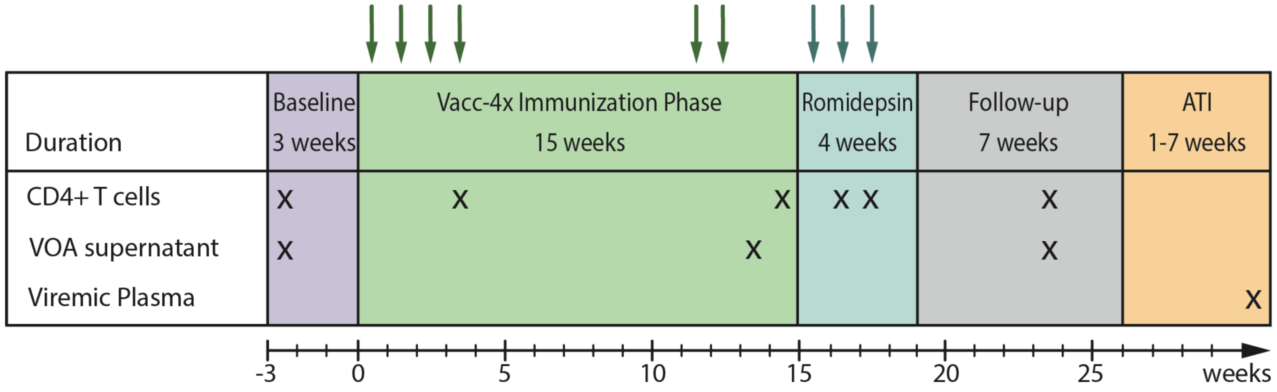

Supplemental Digital Content 1. Outline of study design and sampling time points in the REDUC part B trial. The green arrows indicate administration of Vacc-4x and rhuGM-CSF. The blue arrows indicate administration of romidepsin infusions. ATI, analytical treatment interruption; VOA, viral outgrowth assay.

Supplemental Digital Content 2. Primers and PCR conditions

cDNA synthesis:

Gene specific primers were used to generate cDNA from cell-associated and plasma HIV-1 RNA. For p24-RT we used the PCR 1 reverse primer, listed in the table below. PCR conditions were as follows: 45 °C for 50 minutes, 85 °C for 10 minutes.

P24-RT PCR Conditions:

The p24-RT region was amplified using two rounds of PCR amplification. The primers listed in the table below were used for amplification: PCR1 Forward and PCR1 Reverse for PCR round one, and PCR2 Forward and PCR2 Reverse for PCR round 2. For round 1 of the PCR, the following thermocycler parameters were used: 94 °C for 2 minutes, 94 °C for 30 seconds, 53 °C for 30 seconds, 72 °C for 2 minutes and 30 seconds, 44 cycles of steps 2–4 and 72 °C for 3 minutes. For round 2 of the PCR, the following thermocycler parameters were used: 94 °C for 30 seconds, 50 °C for 30 seconds, 72 °C for 2 minutes and 30 seconds, 41 cycles of steps 1–3 and 72 °C for 3 minutes. The PCR products (2090-2099 kb) representing single HIV-1 sequences were sequenced using Sanger sequencing (Australian Genome Research Facility, Sydney, Australia) with sequencing primers F1-4 and R1-4 as listed in the table below.

Primer Name	Primer Sequence
P24-RT: PCR1 Forward	5’-GCAAGCAGGGARCTAGAACGAT-3’
P24-RT: PCR 1 Reverse	5’-AGTGGTATTACTTCTGTTAGTGCTTT-3’
P24-RT: PCR2 Forward & Sequencing F1	5’-GTCAGCCAAAATTACCCTATAGT-3’
P24-RT: PCR2 Reverse	5’-TTGCCCAATTCAATTTTCCCACTAA-3’
P24-RT: Sequencing F2	5’-ATGACAGAAACCTTGTTGGTCCA-3’
P24-RT: Sequencing F3	5’-TGTTGGAAATGTGGAAAGGAAGGAC-3’
P24-RT: Sequencing F4	5’-ATGGCCCAAAAGTTAAACAATGGC-3’
P24-RT: Sequencing R1	5’-TGGACCAACAAGGTTTCTGTCAT-3’
P24-RT: Sequencing R2	5’-CTGAAGCTCTCTTCTGGTGG-3’
P24-RT: Sequencing R3	5’-TTCTTCTGTCAATGGCCATTGTTTAAC-3’
P24-RT: Sequencing R4	5’-GCTGTCYTTTTCTGGCAG-3’

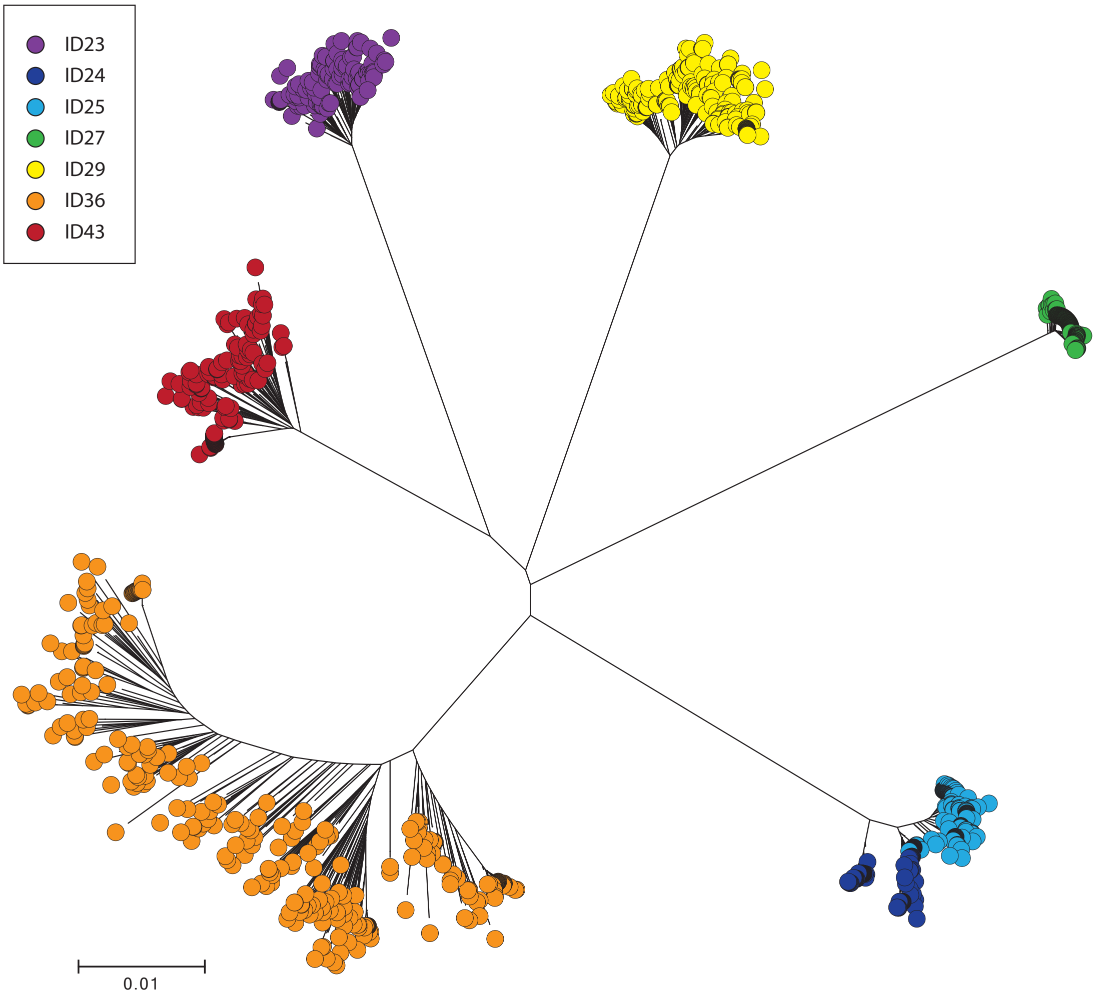

Supplemental Digital Content 3. Neighbour-joining tree of p24-RT of HIV-1 sequences from all participants. Sequences from individual study participants segregate independently except for intermingling of sequences between the known transmission pair ID24 and ID25. Sequences that were classified as defective (containing stop codons or hypermutation) were not included.

Supplemental Digital Content 4. Participant HLA types.

ID	HLA type		
	A	B	C
**23**	02:01/03:01	07:02/18:01	07:01/07:06/07:02
**24**	02:01/03:01	18:01/57:01	05:01/06:02
**25**	02:01/03:01	14:02/44:02	05:01/08:02
**27**	02:06/23:01	35:01/44:03	04:01/04:01
**29**	01:01/68:01	15:01/44:02	03:03/07:04/07:11
**36**	02:01/02:01	07:02/40:01	03:04/07:02
**43**	02:01/03:01	07:02/50:01	06:02/07:02

Supplemental Digital Content 5. Number of intact p24-RT sequences obtained for each sample for each participant in the REDUC part B trial. VOA, viral outgrowth assay; IMM, Immunisation phase; ROM, romidepsin phase; TP, time point; CRNA, cell-associated RNA; PRNA, plasma RNA; - indicates no sample available from this time point.

Participant ID	Baseline DNA	Baseline CRNA	Baseline VOA	IMM TP1 DNA	IMM TP1 CRNA	IMM TP2 DNA	IMM TP2 CRNA	IMM VOA	ROM TP1 DNA	ROM TP1 CRNA	ROM TP2 DNA	ROM TP2 CRNA	Follow-up DNA	Follow-up CRNA	Follow-up VOA	ATI PRNA
**23**	15	0	-	19	2	8	5	-	15	6	16	2	17	3	-	1
**24**	9	0	-	9	0	8	0	-	10	0	7	2	0	1	-	62
**25**	20	0	-	16	2	23	2	-	25	3	14	4	22	2	-	28
**27**	9	6	2	12	3	12	2	1	17	10	12	7	17	2	1	46
**29**	22	21	-	10	13	15	9	2	10	17	7	20	23	17	-	14
**36**	23	12	4	22	23	43	20	1	26	21	35	17	-	-	1	34
**43**	15	2	-	19	0	9	2	1	24	3	20	4	24	2	1	14

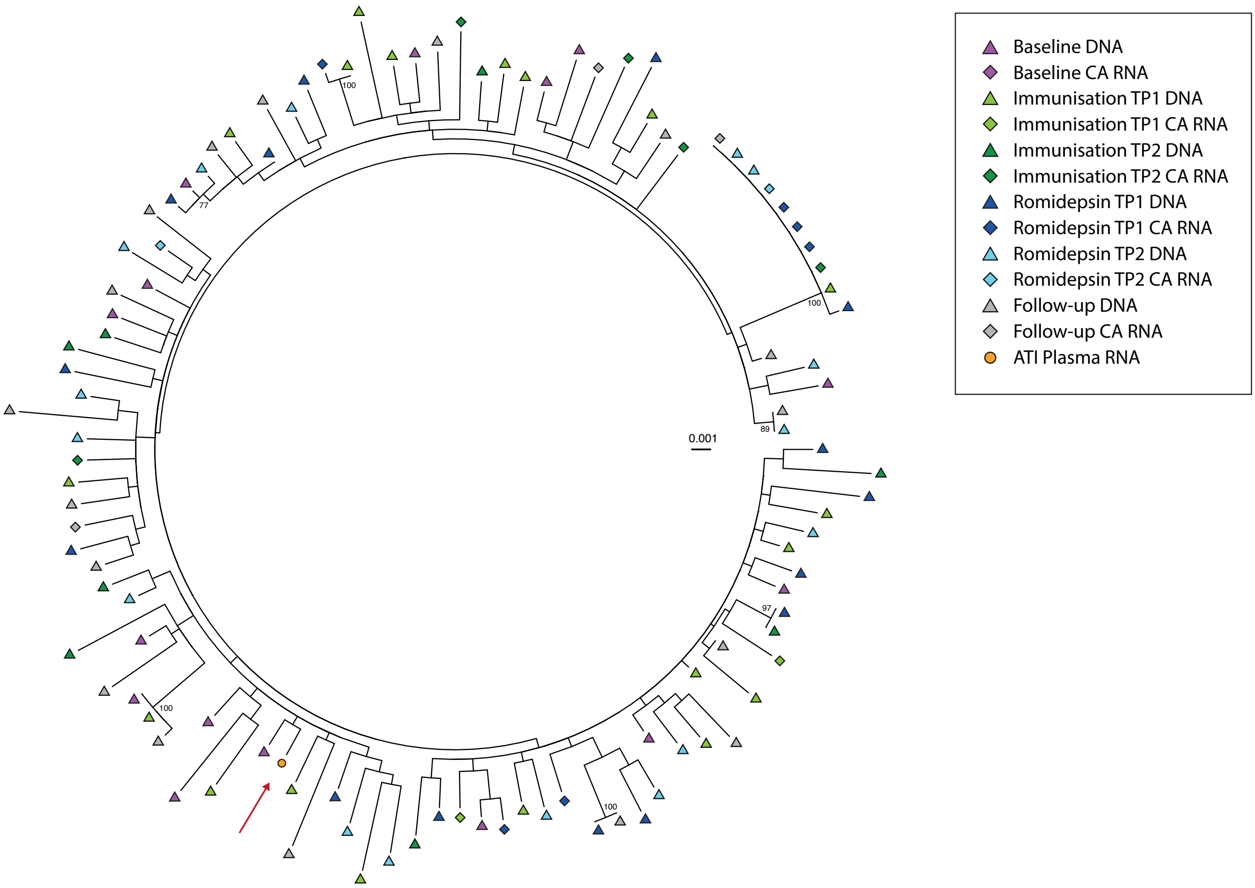

Supplemental Digital Content 6. Maximum likelihood phylogenetic tree of p24-RT of HIV-1 for participant 23. The red arrow indicates an ATI Plasma RNA sequence. CA, cell-associated; TP, time point; ATI, analytical treatment interruption.

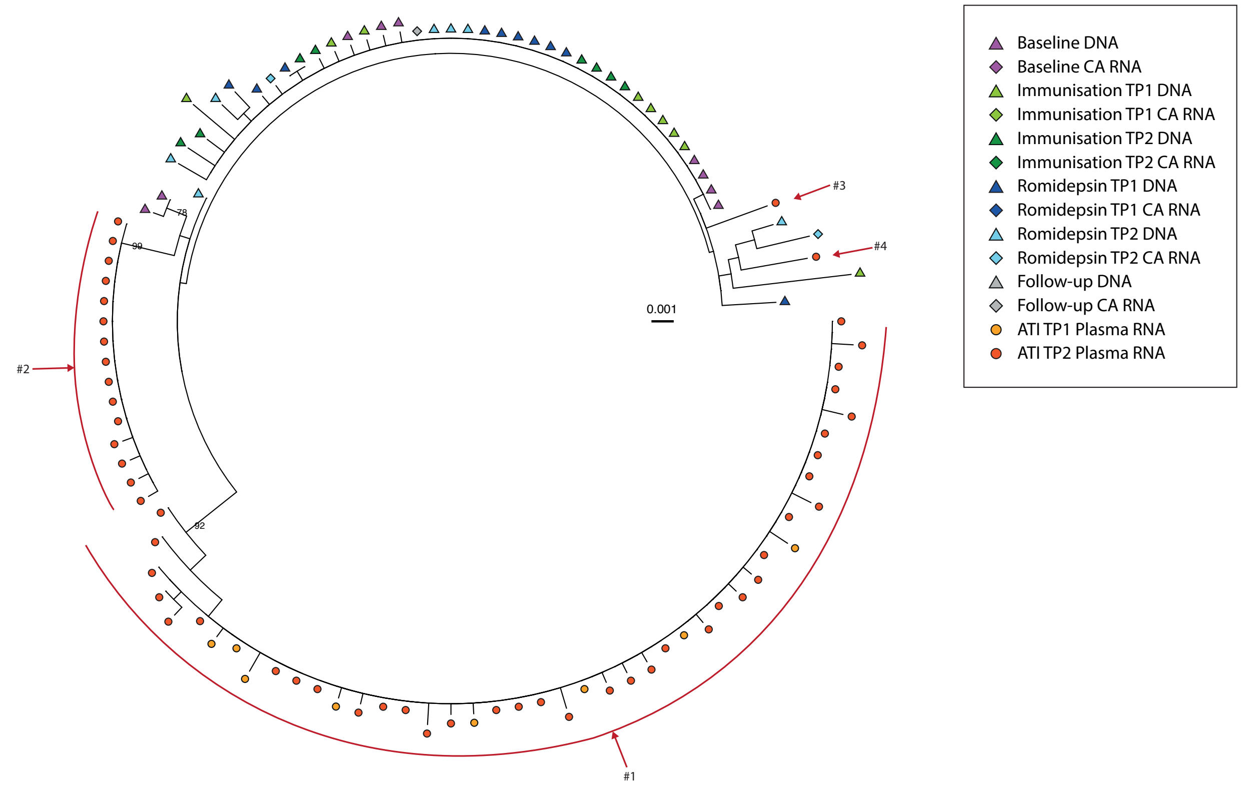

Supplemental Digital Content 7. Maximum likelihood phylogenetic tree of p24-RT of HIV-1 for participant 24. Red arrows indicate ATI Plasma RNA sequences. The numbering of ATI sequences refers to the numbering in Figure 3. CA, cell-associated; TP, time point; ATI, analytical treatment interruption.

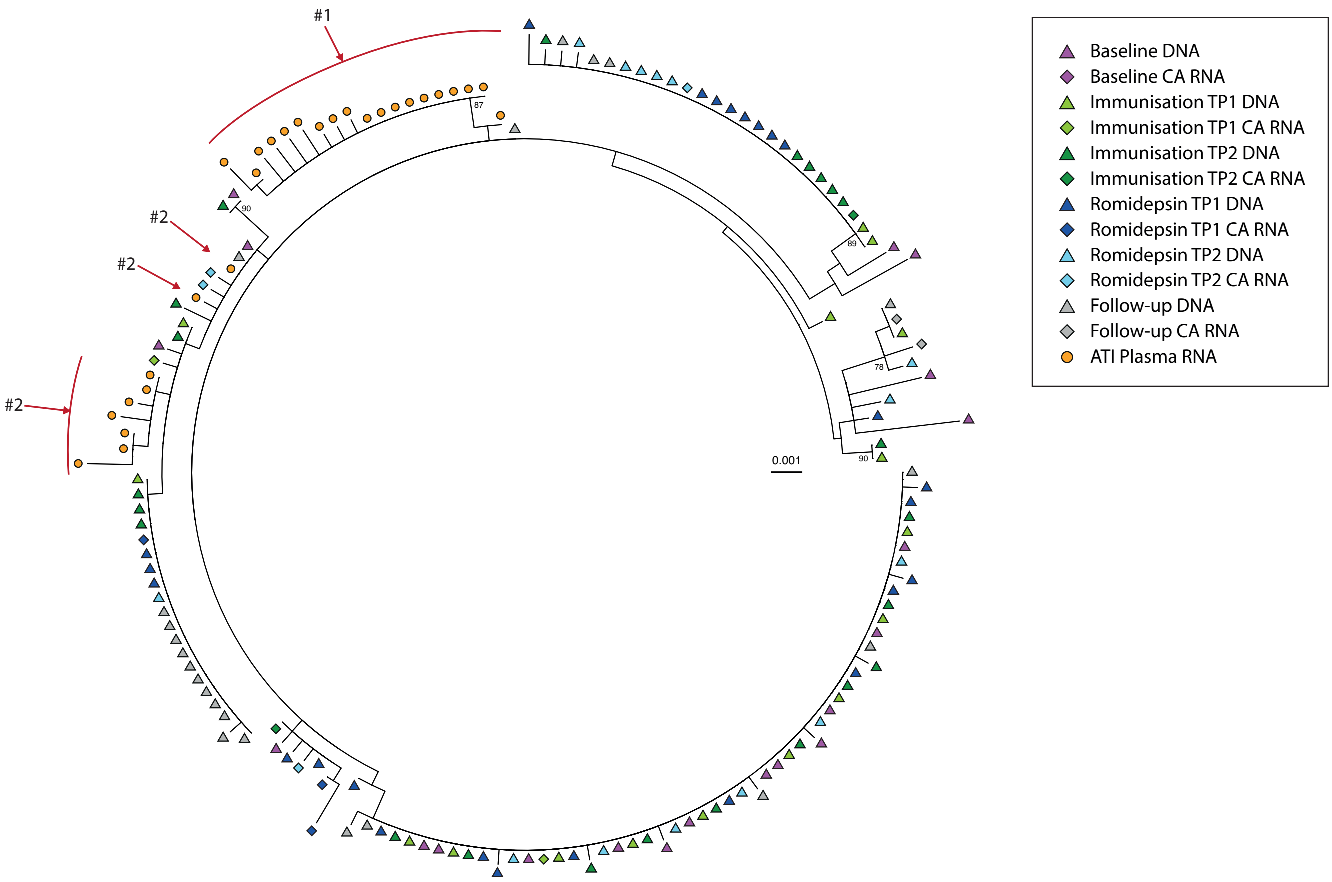

Supplemental Digital Content 8. Maximum likelihood phylogenetic tree of p24-RT of HIV-1 for participant 25. Red arrows indicate ATI Plasma RNA sequences. The numbering of ATI sequences refers to the numbering in Figure 3. CA, cell-associated; TP, time point; ATI, analytical treatment interruption.

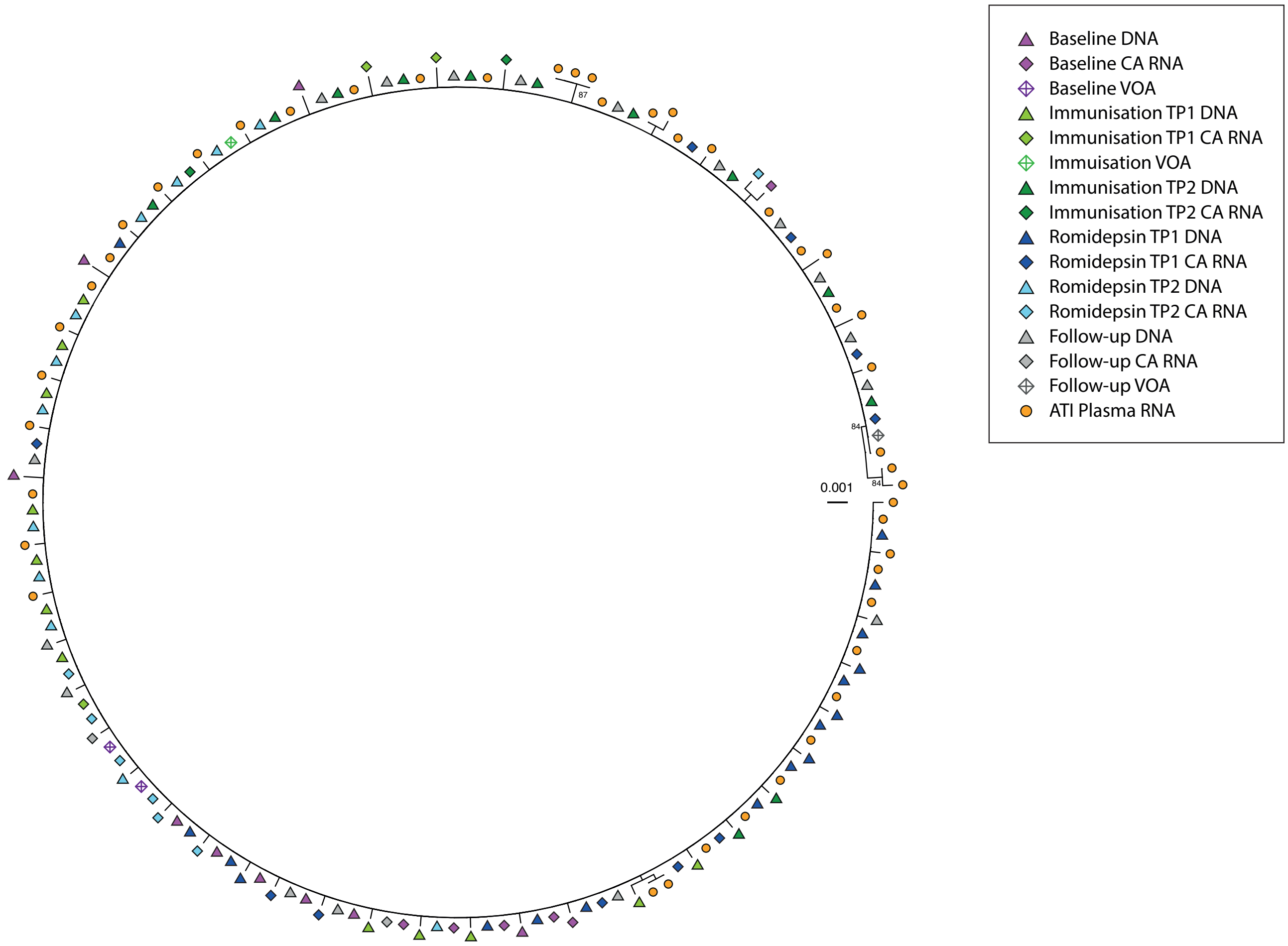

Supplemental Digital Content 9. Maximum likelihood phylogenetic tree of p24-RT of HIV-1 for participant 27. CA, cell-associated; TP, time point; ATI, analytical treatment interruption.

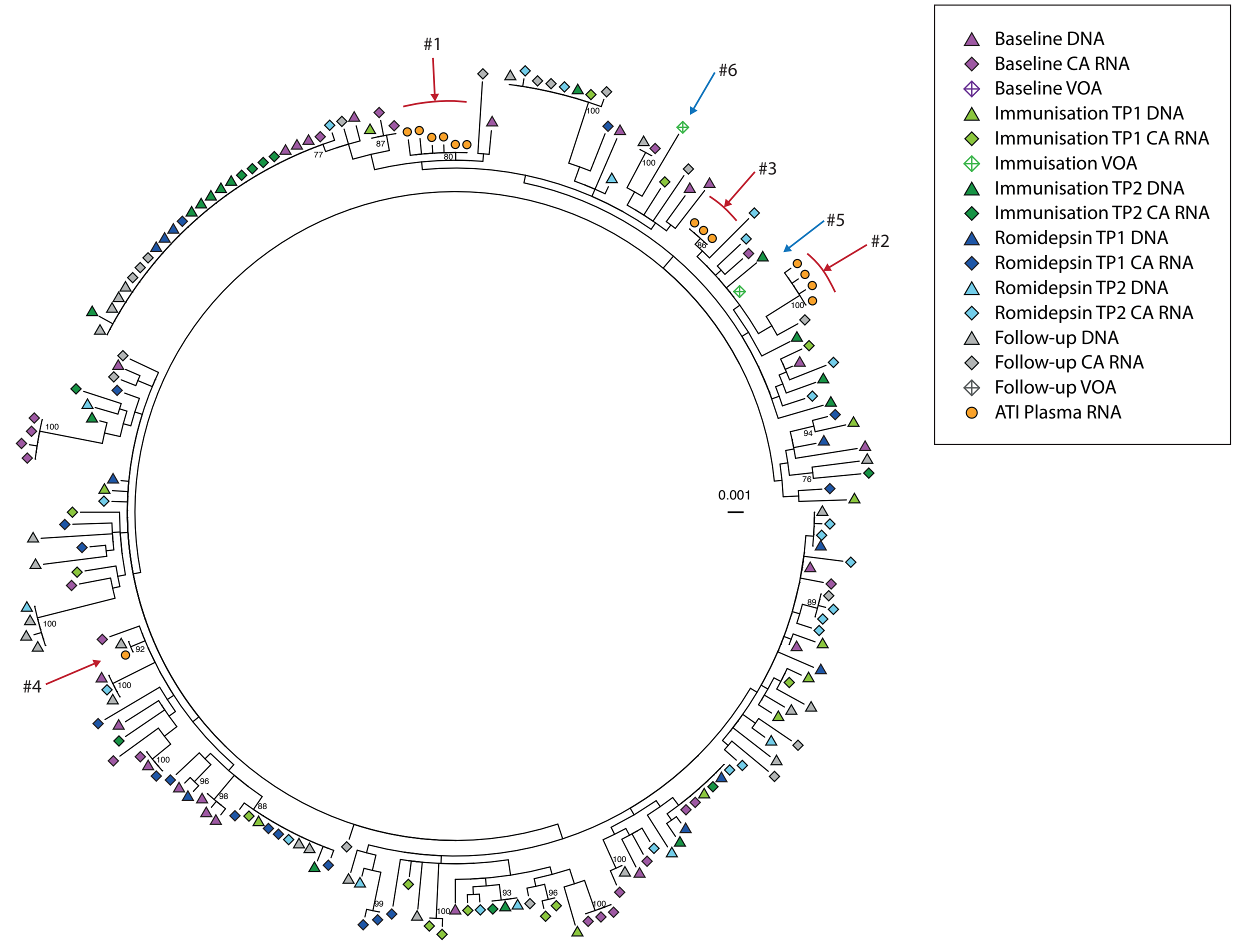

Supplemental Digital Content 10. Maximum likelihood phylogenetic tree of p24-RT of HIV-1 for participant 29. Red arrows indicate ATI Plasma RNA sequences, blue arrows indicate VOA sequences. The numbering of ATI and VOA sequences refer to the numbering in Figure 3. CA, cell-associated; TP, time point; VOA, viral outgrowth assay; ATI, analytical treatment interruption.
